# Expanded Graphite-Modified Melamine–Formaldehyde Adhesive for Fire-Retardant Japanese Cedar Plywood: Physicomechanical and Combustion Performance

**DOI:** 10.3390/polym18141710

**Published:** 2026-07-12

**Authors:** Fang-Yu Hsu, Shan-Ni Yu, Wen-Shao Chang, Jyh-Horng Wu

**Affiliations:** 1 Department of Forestry, National Chung Hsing University, Taichung 402, Taiwan; d113033201@mail.nchu.edu.tw (F.-Y.H.); g112033208@mail.nchu.edu.tw (S.-N.Y.); 2 Lincoln School of Architecture and the Built Environment, University of Lincoln, Lincoln LN6 7TS, UK; wchang@lincoln.ac.uk; 3 Advanced Plant and Food Crop Biotechnology Center, National Chung Hsing University, Taichung 402, Taiwan; 4 The Experimental Forest Management Office, National Chung Hsing University, Taichung 402, Taiwan

**Keywords:** expanded graphite, melamine–formaldehyde resin, Japanese cedar, plywood, fire retardancy, cone calorimeter, pyrolysis gas

## Abstract

Wood-based composite panels are renewable and low-carbon materials, but their inherent flammability limits their broader use in applications requiring improved fire safety. In this study, expanded graphite (EG) was incorporated into a melamine–formaldehyde (MF) adhesive system to fabricate fire-retardant Japanese cedar (*Cryptomeria japonica*) plywood. Two EG types with different expansion ratios, 200-fold expanded graphite (200EG) and 450-fold expanded graphite (450EG), were added to the bonding layer at 10, 20, 30, and 40 phr. The effects of EG expansion ratio and content on the physicomechanical properties, combustion behavior, char morphology, and pyrolysis gas evolution of plywood were investigated. EG had limited effects on air-dried density and moisture content, but increasing EG content reduced bonding shear strength and parallel-direction flexural properties, particularly in the 450EG series. Cone calorimeter analysis showed that EG reduced the heat release rate and total heat release by promoting the formation of an expanded carbonaceous barrier layer. Among the tested formulations, 30 phr 200EG provided the most favorable balance between mechanical performance and fire-retardant efficiency, showing a visually more continuous char coverage while maintaining acceptable bonding properties. High-temperature furnace–FTIR analysis indicated that excessive EG loading or high-expansion-ratio EG increased CO_2_ and CO-related signals at 400–700 °C, probably because of a visually looser char morphology, structural disruption during expansion, and gas release from EG intercalating agents. Although the tested panels did not fully meet the CNS Grade 3 fire-retardant requirement, the results provide a useful basis for the modification and performance optimization of halogen-free fire-retardant wood-based composite panels.

## 1. Introduction

The development of sustainable, low-carbon, and resource-efficient materials has become an important objective in modern materials science and building technology. Wood is a renewable lignocellulosic material with carbon storage capacity, low embodied energy, favorable processability, and a high strength-to-weight ratio. These advantages have supported the widespread use of wood and wood-based composites in construction, interior decoration, furniture, and engineered panel products [1,2,3]. In Taiwan, the utilization of domestic timber has remained limited since the implementation of the logging ban on natural forests in 1991, and the forest products industry continues to rely heavily on imported wood resources. Therefore, increasing the value-added use of domestic plantation timber is important for forest resource management and wood-based materials development. Among locally available species, Japanese cedar (*Cryptomeria japonica*) is a promising candidate for wood composite applications because of its relatively low density, low drying shrinkage, and good workability [4,5]. Converting Japanese cedar veneers into functional plywood may therefore improve the utilization efficiency and market value of domestic timber resources.

Plywood is one of the most widely used wood-based composite panels. Its cross-laminated veneer structure provides improved dimensional stability, balanced mechanical properties, and favorable processability compared with solid wood. Nevertheless, like solid wood, plywood remains inherently combustible because its main constituents (cellulose, hemicellulose, and lignin) undergo thermal decomposition and release flammable volatiles when exposed to heat or flame. During combustion, untreated plywood may generate high heat release, rapid flame spread, smoke production, and structural degradation, which restricts its use in applications requiring fire safety performance. Therefore, enhancing the fire retardancy of plywood while maintaining acceptable mechanical and bonding performance is essential for expanding the use of wood-based panels in building and interior applications. Although halogen-containing fire retardants can provide effective flame inhibition, concerns regarding toxic, corrosive, and environmentally persistent combustion products have promoted the development of halogen-free systems [6,7]. Among these, intumescent and carbon-based additives are particularly attractive because they promote char formation and reduce heat and mass transfer during combustion.

Expanded graphite (EG) is a halogen-free carbonaceous intumescent material derived from natural flake graphite through intercalation treatment [8,9]. Upon heating, the intercalated compounds between the graphite layers decompose and release gases, causing separation and rapid expansion of the graphite layers. This process produces a worm-like expanded carbonaceous structure that can act as a physical protective barrier, thereby suppressing heat transfer, oxygen diffusion, and the release of combustible volatiles [10,11,12]. Compared with non-intumescent carbonaceous fillers, such as graphite powder, carbon black, or carbon fibers, the main advantage of EG is its ability to actively expand during combustion and form a continuous carbonaceous protective layer. On the other hand, inorganic or ceramic additives such as silicon carbide can act as heat-resistant or thermal-insulating fillers and improve thermal protection under high heat flux; however, their mechanisms are mainly associated with thermally stable filler or ceramic-reinforcing effects, which differ from the fire-retardant mechanism of EG that actively expands during combustion and forms a carbonaceous protective layer [13]. Therefore, EG can be regarded as a promising carbonaceous intumescent additive for fire-retardant modification of wood-based materials, and its fire-retardant effect mainly arises from the physical barrier formed by thermal expansion.

Although EG has been widely investigated in polymer matrices, coatings, foams, and wood-based composites, its incorporation into the thermosetting adhesive layer of plywood remains relatively limited [12,14,15]. In plywood, the adhesive layer not only bonds adjacent veneers but also influences stress transfer, interfacial integrity, moisture resistance, and thermal response. Incorporating EG into melamine–formaldehyde (MF) adhesive may therefore provide a feasible strategy for producing fire-retardant plywood without fully impregnating the wood veneer [16]. However, EG may also alter adhesive viscosity, wetting behavior, veneer penetration, and interfacial continuity, thereby affecting bonding strength, flexural properties, and fire-retardant efficiency. In this study, Japanese cedar veneers and MF resin were used to prepare EG-modified plywood. Two types of EG with different expansion ratios, 200EG and 450EG, were incorporated into the MF adhesive at different loading levels. The objectives were to evaluate the effects of EG expansion ratio and content on plywood physicomechanical properties, heat release behavior, char-layer morphology, and pyrolysis gas evolution using cone calorimetry and high-temperature furnace–FTIR analysis. This study provides a basis for optimizing adhesive-based, halogen-free fire-retardant treatments for Japanese cedar plywood.

## 2. Materials and Methods

### 2.1. Materials

Rotary-cut Japanese cedar veneers were purchased from Wand Tsai Industrial Co. (Chiayi, Taiwan). The veneer thickness was 3.3 ± 0.3 mm. Melamine–formaldehyde resin (MF-600; solid content: 61%; viscosity: 530 mPa·s; curing temperature: 120 °C) was supplied by Wood Glue Industrial Co. (Tainan, Taiwan) and used as the thermosetting adhesive matrix. Expanded graphite (EG) was supplied by Homytech Co. (Taoyuan, Taiwan) and used as the halogen-free intumescent fire-retardant additive. Two types of EG were selected to evaluate the influence of expansion ratio and particle size. The first type, G36E-80-200, had a nominal particle size of 80 mesh and an expansion ratio of approximately 200-fold; this material is referred to as 200EG. The second type, G36E-50-450, had a nominal particle size of 50 mesh and an expansion ratio of approximately 450-fold; this material is referred to as 450EG. The particle size and expansion ratio of both EG types were provided by the supplier. Prior to plywood fabrication, the veneers were conditioned at 20 °C and 65% relative humidity (RH). All materials were used as received without further purification.

### 2.2. Preparation of EG-Modified Plywood

EG-modified plywood was prepared by incorporating different amounts of EG into the MF adhesive before veneer assembly. The EG contents were set at 10, 20, 30, and 40 phr, based on the solid content of the MF resin. Plywood without EG was prepared as the control group. Because the two EG types differed in particle size and expansion behavior, preliminary trials were conducted to determine suitable adhesive spread rates for each EG series. The selection was based on the EG coverage ratio in the adhesive layer, as shown in Supplementary Table S1 and Figure S1, and the corresponding bonding shear strength, as summarized in Supplementary Table S2. Based on these results, the MF resin spread rates were set at 300 g/m^2^ for the 200EG groups and 350 g/m^2^ for the 450EG groups to ensure adequate veneer surface coverage while maintaining acceptable bonding performance. Because different adhesive spread rates were used for the 200EG and 450EG series, EG-free control panels were prepared separately for each series and are denoted as 200EG_0_ and 450EG_0_, respectively. Therefore, comparisons between the 200EG and 450EG series should be interpreted under the present processing conditions, in which the adhesive spread rate was adjusted according to the EG type to ensure adequate veneer surface coverage. For each formulation, the predetermined amount of EG was gradually added to the MF resin and mixed at 100 rpm for 5 min to obtain an EG-containing adhesive mixture. The adhesive was then applied to one side of the veneer. Three veneer layers were assembled with adjacent grain directions perpendicular to each other to form a cross-laminated plywood structure. Spacers were used to control the final panel thickness at approximately 8 mm. The assembled mats were hot-pressed at 150 °C for 10 min, followed by cold pressing for 10 min. After pressing, plywood panels with dimensions of 600 mm × 600 mm × 8 mm and 300 mm × 300 mm × 8 mm were prepared and cut into specimens for subsequent physicomechanical, morphological, thermal, and fire performance analyses.

### 2.3. Physicomechanical Characterization

The physicomechanical properties of the plywood specimens were evaluated in terms of air-dried density, moisture content, bonding shear strength, modulus of rupture (MOR), and modulus of elasticity (MOE). Prior to testing, all specimens were conditioned at 20 °C and 65% RH until a constant mass was reached. Air-dried density and moisture content were determined according to CNS 1349 [17]. Specimens with dimensions of 75 mm × 75 mm × 8 mm were used for these measurements, and air-dried density and moisture content were calculated using Equations (1) and (2), respectively. In these equations, m_0_ represents the oven-dry mass (kg) of the specimen, m_1_ represents the air-dried mass (kg) of the specimen after conditioning at 20 °C and 65% RH, and V represents the air-dried volume (m^3^) of the specimen. Bonding shear strength was also determined according to CNS 1349 [17] using a Shimadzu Autograph AG-X universal testing machine (Kyoto, Japan). In brief, specimens with dimensions of 75 mm × 25 mm × 8 mm were immersed in warm water at 60 ± 3 °C for 3 h, cooled in cold water to room temperature, and then tested at a loading rate of 5880 N/min. Bonding shear strength was calculated using Equation (3), where p_s_ represents the maximum load (N) in the bonding shear test, b represents the specimen width (mm), and s represents the distance (mm) between the saw grooves on the bonding surface. Flexural properties were determined according to CNS 11671 [18] using the same universal testing machine. The MOR and MOE of specimens with dimensions of 240 mm × 50 mm × 8 mm were measured using a three-point bending test at a loading rate of 14.7 MPa/min and a span of 24 times the specimen thickness. The tests were conducted in both directions, with the face-grain direction parallel and perpendicular to the span direction. MOR and MOE were calculated using Equations (4) and (5), respectively. In these equations, *P* represents the maximum load (N), *L* represents the span length (mm), Δ*P* represents the load difference within the proportional limit (N), Δ*Y* represents the corresponding deflection (mm), *b* represents the specimen width (mm), and *h* represents the specimen thickness (mm). Eleven replicate specimens were used for the air-dried density, moisture content, and bonding shear strength tests, whereas thirteen replicate specimens were used for the flexural tests.

(1) $$\mathrm{Density}\;\left(kg/m^{3}\right)=m_{1}/V$$

(2) $$\mathrm{Moisture}\;\mathrm{content}\;(\%)\;=\left(m_{1}-m_{0}\right)/m_{0}\;\times \;100$$

(3) $$\mathrm{Bonding}\;\mathrm{shear}\;\mathrm{strength}\;(\mathrm{MPa})\;=p_{s}/\mathrm{bs}$$

(4) $$\mathrm{MOR}\;(\mathrm{MPa})\;=\;3PL/2bh^{2}$$

(5) $$\mathrm{MOE}\;(\mathrm{MPa})\;=∆PL^{3}/4∆Ybh^{3}$$

### 2.4. Scanning Electron Microscopy

The morphology of the fractured bonding interfaces was observed using a Hitachi TM3000 tabletop scanning electron microscope (Tokyo, Japan). Specimens collected after the bonding shear test were used to examine the distribution of EG particles, the continuity of the adhesive layer, and the presence of voids or interfacial defects. SEM images were obtained under low-vacuum conditions at an accelerating voltage of 15 kV.

### 2.5. High-Temperature Furnace–FTIR Analysis

The evolution of pyrolysis gases during thermal decomposition was analyzed using a high-temperature furnace coupled with Fourier transform infrared (FTIR) spectroscopy. A Nabertherm LHT 04/17 SW high-temperature furnace (Lilienthal, Germany) was connected to a PerkinElmer Spectrum 100 FTIR spectrometer (Waltham, MA, USA). Specimens with dimensions of 30 mm × 30 mm × 8 mm were placed in a crucible and heated under a nitrogen atmosphere. The temperature was increased from 40 °C to 800 °C at a heating rate of 10 °C/min. Nitrogen was supplied at a flow rate of 100 L/h. FTIR spectra of the released gases were recorded over 80 min at a resolution of 4 cm^−1^ with 32 scans per spectrum. The spectral range was 4000–650 cm^−1^. The major absorption bands associated with H_2_O, CO_2_, CO, C–H, C=O, C–O, C–O–C, NH_3_, sulfate-related species, and other pyrolysis products were analyzed to clarify the thermal decomposition behavior of untreated veneer and EG-modified plywood.

### 2.6. Cone Calorimeter Analysis

The fire-retardant properties of the plywood specimens were evaluated using a cone calorimeter according to CNS 14705-1:2019 [19]. A Deatak CC-2 cone calorimeter (McHenry, IL, USA) was used. Specimens with dimensions of 100 mm × 100 mm × 8 mm were conditioned at 23 °C and 50% RH until a constant mass was reached. Before testing, the sides and bottom of each specimen were wrapped with aluminum foil to ensure controlled exposure of the top surface. The cone calorimeter test was conducted at a radiant heat flux of 50 kW/m^2^ and a gas flow rate of 0.024 ± 0.002 m/s. The combustion duration was 300 s. The heat release rate (HRR), peak heat release rate (HRR_peak_), and total heat release within 300 s (THR_300s_) were recorded. HRR and THR were calculated using Equations (6) and (7), respectively. Three replicate specimens were tested for each formulation. After combustion, the residual char morphology was photographed and compared to evaluate the surface coverage and apparent continuity of the EG-derived char layer.

(6) $$\mathrm{HRR}\;(\mathrm{kW}/m^{2})\;=\left(∆h_{c}/r_{0}\right)\;\left(1.10\right)\;C{\;\left(∆p/Te\right)}^{1/2}\left(X_{O_{2}}^{0}-X_{O_{2}}\right)/\left(1.105-1.5X_{O_{2}}\right)=\dot{q}\left(t\right)$$

(7) $$\mathrm{THR}\;(MJ/m^{2})=10^{-3}\int_{0}^{t_{f}}\dot{q}\left(t\right)\;dt$$

where HRR is the heat release rate per unit area at time *t* during combustion (kW/m^2^); Δ*hc*/r_0_ is the heat released per unit oxygen consumption (kJ/kg O_2_); *C* is the system calibration constant; Δ*p* is the pressure difference across the orifice plate; *Te* is the exhaust gas temperature (K); $X_{O_{2}}^{0}$ is the ambient oxygen volume fraction; and $X_{O_{2}}$ is the oxygen volume fraction after combustion. THR is the total heat release during combustion (MJ/m^2^).

### 2.7. Analysis of Variance

All experimental results are expressed as the mean ± standard deviation. Statistical differences among groups were analyzed using one-way analysis of variance followed by Scheffe’s post hoc test. Differences were considered statistically significant at *p* < 0.05.

## 3. Results and Discussion

### 3.1. Effects of EG Expansion Ratio and Content on the Physicomechanical Properties of Plywood

The physicomechanical properties of plywood prepared with different EG expansion ratios and contents are summarized in Table 1. The density of the 200EG plywood groups ranged from 478 to 534 kg/m^3^, whereas that of the 450EG groups ranged from 533 to 608 kg/m^3^. The moisture contents of the 200EG and 450EG groups were 8.5–9.9% and 9.0–10.5%, respectively. Overall, the addition of EG did not produce a consistent increasing or decreasing trend in density or moisture content. All plywood panels satisfied the density range of 400–800 kg/m^3^ and the moisture content requirement of less than 14% specified in CNS 1349 for ordinary plywood [17]. These results indicate that incorporating EG into the MF adhesive layer did not substantially alter the basic physical properties of the plywood panels.

In contrast to density and moisture content, bonding shear strength was clearly affected by EG incorporation. As shown in Table 1, the average bonding shear strength of the 200EG group decreased from 799 kPa for the control plywood to 538 kPa for plywood containing 40 phr EG. A similar but more pronounced reduction was observed in the 450EG group, in which the bonding shear strength decreased from 799 kPa to 470 kPa as EG content increased to 40 phr. The minimum bonding shear strength also decreased with EG addition, particularly in the 450EG group, where the lowest value reached 253 kPa. These results demonstrate that excessive EG loading can negatively affect adhesive bonding performance, especially when EG with a higher expansion ratio and larger particle size is used. It should be noted that the comparison between the 200EG and 450EG series was made under the present processing conditions, in which different adhesive spread rates were used to accommodate the different particle sizes and expansion behaviors of EG. Therefore, the more pronounced reduction observed in the 450EG series should be interpreted as the combined effect of EG characteristics and the corresponding processing conditions, rather than solely as an effect of expansion ratio. The reduction in bonding performance can be attributed to changes in the wood–polymer adhesive interphase. The formation of a strong plywood bond requires adequate adhesive wetting, penetration into veneer surface pores and cell lumens, and the development of a continuous cured adhesive network [20]. The addition of EG increases the inorganic filler fraction in the MF adhesive and increases viscosity (Supplementary Table S3), which may reduce flowability and limit adhesive penetration into the wood substrate. At higher EG contents, particle agglomeration may also occur, leading to discontinuous adhesive distribution and reduced effective contact area between adjacent veneers. These effects weaken mechanical interlocking and stress transfer across the bonding layer.

**Figure 1:**
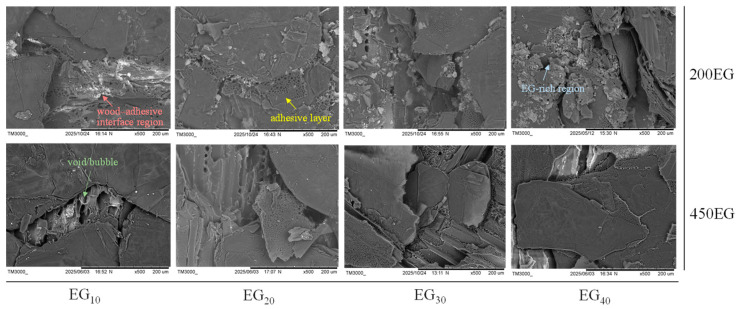
SEM micrographs of fractured bonding interfaces of EG-modified plywood after bonding shear testing. Arrows indicate representative wood–adhesive interfacial regions, adhesive regions, voids/bubbles, and a representative EG-rich region. The images show the influence of EG expansion ratio and content on adhesive-layer continuity, interfacial morphology, and void formation.

SEM observations of the fractured bonding interfaces further revealed changes in EG distribution, adhesive-layer continuity, and interfacial morphology. As shown in Figure 1, the EG-containing adhesive layer exhibited voids, bubbles, and discontinuous regions, indicating that EG incorporation affected the compactness and continuity of the cured MF adhesive network. These features became more pronounced with increasing EG content and were particularly evident in the 450EG series. Under the present processing conditions, the more pronounced interfacial defects observed in the 450EG series may be associated with the combined effects of its larger nominal particle size, higher expansion capacity, and the corresponding adhesive spread conditions. These factors may have promoted local EG accumulation within the bonding layer and affected the uniform distribution of the adhesive phase. As a result, the effective contact area between adjacent veneers was reduced, and interfacial stress transfer across the bonding layer was weakened. During shear loading, these discontinuous regions and interfacial defects could act as stress concentration sites, leading to premature failure and reduced bonding strength. Similar filler-size-related effects on composite performance have been reported previously [21,22]. Therefore, although EG provides fire-retardant functionality, uniform EG dispersion, good compatibility with the MF adhesive matrix, and preservation of adhesive-layer continuity are essential for maintaining interfacial integrity and plywood bonding performance.

The flexural properties also depended on EG type and content. As shown in Table 1, in the 200EG group, the parallel-direction MOR (MOR_//_) decreased from 63 MPa in the control group to 45 MPa at 40 phr EG, whereas the parallel-direction MOE (MOE_//_) decreased only slightly from 7.4 to 7.0 GPa. In the 450EG group, the reduction was more severe: the MOR_//_ decreased from 56 to 26 MPa, and the MOE_//_ decreased from 7.4 to 3.8 GPa as EG content increased to 40 phr. These results indicate that, under the present processing conditions, the 450EG series had a stronger adverse effect on flexural performance. This effect should be interpreted as the combined influence of the larger nominal particle size, higher expansion ratio, adjusted adhesive spread rate, and resulting adhesive-layer continuity, rather than solely as the effect of EG expansion ratio. This trend is consistent with previous findings showing that the incorporation of expandable graphite into polymer composites may reduce mechanical properties when filler dispersion and interfacial compatibility are insufficient [23]. The decline in parallel-direction flexural properties can be explained by the weakened bonding layer and reduced interfacial integrity. During bending, stress transfer between veneers depends strongly on the continuity and strength of the adhesive layer. When excessive EG particles are present, the cured MF adhesive network may become less continuous, and the wood–adhesive interface may contain more defects. These defects reduce the ability of the panel to distribute bending stress, resulting in lower MOR and MOE. In contrast, the perpendicular-direction MOR (MOR_⊥_) and MOE (MOE_⊥_) were less sensitive to EG content. The MOR_⊥_ and MOE_⊥_ values of the 200EG plywood ranged from 9 to 12 MPa and 0.7 to 0.8 GPa, respectively, whereas those of the 450EG plywood ranged from 12 to 15 MPa and 0.7 to 1.0 GPa, respectively. The slightly higher MOR_⊥_ in some 450EG groups may be associated with their relatively higher panel density. Overall, the results indicate that EG incorporation mainly compromised bonding shear strength and parallel-direction bending performance, whereas perpendicular-direction flexural properties were less affected.

### 3.2. Effects of EG on Heat Release Behavior and Fire-Retardant Performance

**Figure 2:**
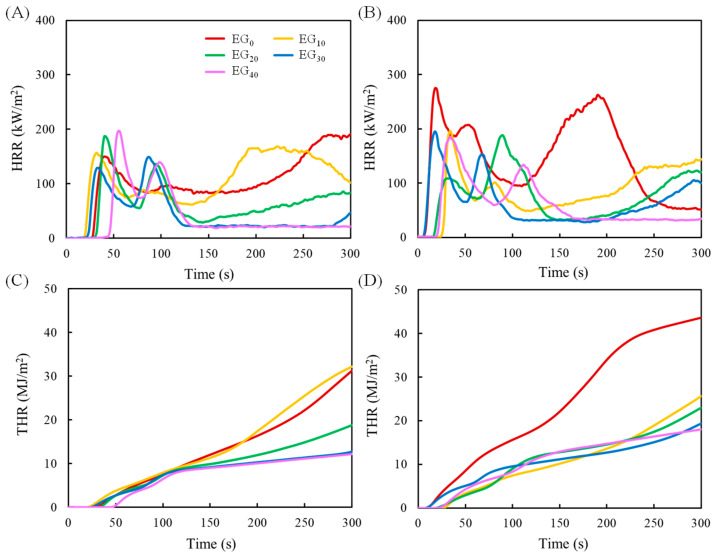
Heat release behavior of EG-modified plywood measured by cone calorimetry: HRR curves of (**A**) 200EG and (**B**) 450EG plywood, and THR curves of (**C**) 200EG and (**D**) 450EG plywood.

The fire-retardant performance of the EG-modified plywood was evaluated using cone calorimetry. As shown in Figure 2A and Table 2, the HRR curves of the 200EG plywood generally exhibited two main heat-release peaks. The first peak was associated with the combustion of the exposed surface veneer, whereas the second peak was related to heat transfer into the inner veneer layer and subsequent combustion of the internal wood structure. In the control and low-EG-content groups, an additional heat-release peak appeared during the later stage of combustion. This third peak may have resulted from cracking or structural failure of the charred surface layer, which exposed the bottom veneer to heat and oxygen and induced renewed combustion. As EG content increased, the HRR curves decreased markedly during the later combustion stage. In the 200EG group, THR_300s_ decreased from 32.7 MJ/m^2^ for the control plywood to 13.0 MJ/m^2^ for plywood containing 40 phr EG. The 200EG_30_ group showed a low THR_300s_ of 12.9 MJ/m^2^ and a reduced HRR_peak_ of 157 kW/m^2^. These results indicate that 200EG effectively suppressed heat release, particularly at 30–40 phr. The reduction in heat release is mainly attributed to the intumescent action of EG. Upon heating, EG expands and forms a carbonaceous layer that covers the plywood surface. This expanded char layer reduces heat transfer to the underlying wood, limits oxygen diffusion, and suppresses the escape of combustible volatiles [10,11,12,24].

A similar improvement in fire-retardant performance was observed in the 450EG group. As shown in Figure 2B,D and Table 2, THR_300s_ decreased from 46.2 MJ/m^2^ in the control plywood to 15.1 and 16.5 MJ/m^2^ in the 450EG_30_ and 450EG_40_ groups, respectively. The HRR_peak_ values of the EG-containing 450EG plywood were also significantly lower than those of the control group. These results confirm that EG can effectively reduce the heat release of plywood in both EG series, which is consistent with previous reports on EG-containing wood-based composites [24]. However, the detailed combustion behavior differed between the two EG types. Because different adhesive spread rates were applied to the 200EG and 450EG series, the observed differences in combustion behavior should be interpreted as the combined effects of EG expansion ratio, particle size, adhesive distribution, and panel manufacturing conditions. Under the present processing conditions, 450EG did not provide a clear advantage over 200EG in balancing heat-release suppression and apparent char-layer continuity. At low EG contents, the 450EG plywood still exhibited distinct later-stage heat-release peaks. This behavior may be associated with the more intense expansion of high-expansion-ratio EG during the early stage of combustion. Rapid volumetric expansion can disrupt the surface veneer and char layer, resulting in cracks and pathways for heat and oxygen penetration. Although 450EG may undergo more intense expansion, the resulting char surface appeared visually less uniform than that formed by 200EG. As a result, the barrier effect may have been less continuous, particularly when the EG content was insufficient to provide adequate surface coverage.

**Figure 3:**
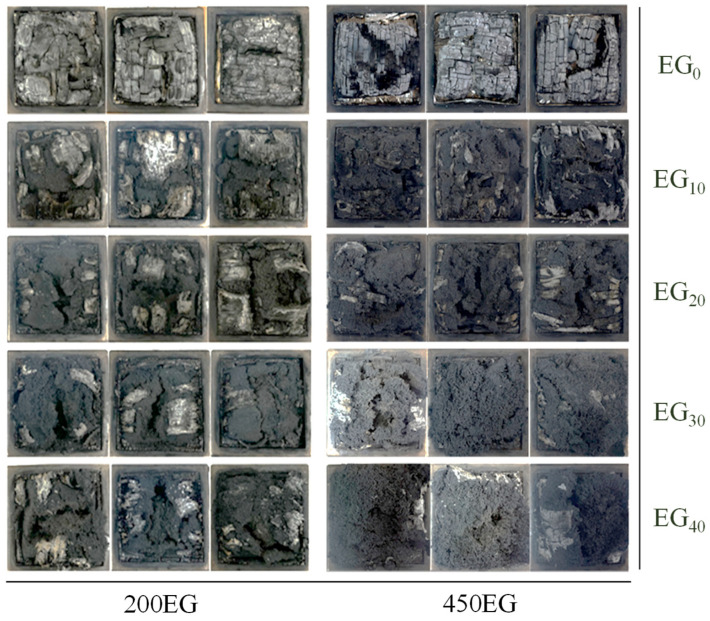
Post-combustion char morphology of plywood containing different EG expansion ratios and contents after cone calorimeter testing. The rows correspond to EG_0_, EG_10_, EG_20_, EG_30_, and EG_40_, and the columns correspond to the 200EG and 450EG series.

The post-combustion char morphology provides visual support for the fire-retardant mechanism. As shown in Figure 3, the control specimens were largely converted to ash residues, and burn-through was observed, indicating that untreated plywood could not form an effective protective barrier. With increasing EG content, the residual char gradually changed from a fragmented and discontinuous structure to a more complete expanded layer. The EG_30_ and EG_40_ groups showed greater surface coverage and more continuous char appearance. This observation suggests that an appropriate EG loading promoted the formation of a protective intumescent char layer [24,25]. Nevertheless, the residual char surface of the 450EG groups appeared visually looser and more uneven than that of the 200EG groups. This difference may be related to the higher expansion ratio of 450EG, which could cause more intense expansion and a less uniform char appearance. Such a visually uneven char surface may still contribute to heat insulation, but it may also create discontinuous regions that allow heat and volatile products to pass through more easily. In comparison, 200EG_30_ exhibited a more visually continuous char surface, which was consistent with its lower heat release and acceptable mechanical properties. Although 200EG_40_ showed a THR_300s_ value comparable to that of 200EG_30_, the additional EG loading did not provide a clear further reduction in heat release and may increase the risk of adhesive-layer discontinuity; therefore, 200EG_30_ was considered the more balanced formulation. According to CNS 14705-1 [19], Grade 3 fire-retardant materials for interior finishing applications require an HRR below 200 kW/m^2^ within 300 s, with the duration above this value not exceeding 10 s, and a THR not exceeding 8 MJ/m^2^. None of the plywood formulations in this study fully satisfied these requirements, mainly because the THR values remained higher than the standard limit. However, EG addition substantially reduced THR and improved char formation compared with the control plywood. Among the tested formulations, 200EG_30_ provided the most balanced performance, combining reduced heat release, more continuous apparent char coverage, and acceptable mechanical properties. Therefore, future studies may use this formulation as a reference and further improve the overall fire-retardant performance by enhancing the fire-retardant efficiency of the adhesive layer, incorporating fire-retardant-treated veneers, optimizing the veneer layup configuration, and combining EG with other halogen-free fire retardants to further reduce the total heat release while maintaining acceptable mechanical performance.

### 3.3. Pyrolysis Gas Evolution During Thermal Decomposition

**Figure 4:**
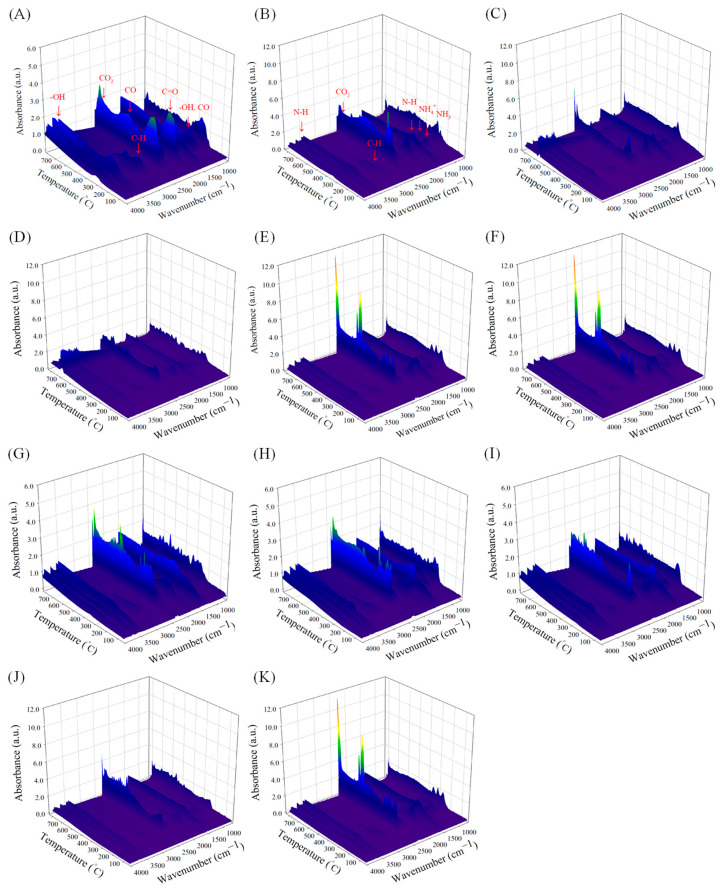
High-temperature furnace–FTIR spectra of pyrolysis gases released from untreated veneer and EG-modified plywood: (**A**) untreated veneer; (**B**) 200EG_0_; (**C**) 200EG_10_; (**D**) 200EG_20_; (**E**) 200EG_30_; (**F**) 200EG_40_; (**G**) 450EG_0_; (**H**) 450EG_10_; (**I**) 450EG_20_; (**J**) 450EG_30_; and (**K**) 450EG_40_.

High-temperature furnace–FTIR analysis was conducted to clarify the effects of EG on the thermal decomposition behavior and gas evolution of plywood. As shown in Figure 4A, untreated Japanese cedar veneer showed an O–H absorption band at 3300–3500 cm^−1^ at approximately 100 °C, corresponding mainly to moisture evaporation. As the temperature increased to 200–350 °C, characteristic absorption bands appeared at 2810–2950 cm^−1^, 2350 cm^−1^, 2110–2170 cm^−1^, 1700–1850 cm^−1^, 1400 cm^−1^, and 900–1200 cm^−1^. These bands correspond to C–H, CO_2_, CO, C=O, C–H deformation, and C–O–C vibrations, respectively, and indicate the decomposition of cellulose, hemicellulose, and lignin with the release of volatile products [26]. Compared with untreated veneer, plywood without EG exhibited additional absorption bands associated with the MF adhesive. As shown in Figure 4B,G, peaks at 3400–3450 cm^−1^, 1580 cm^−1^, and 930–970 cm^−1^ were attributed to N–H, C=N/N–H, and NH_3_-related signals, respectively. These bands suggest that the MF resin underwent deamination and network cleavage during heating, releasing nitrogen-containing non-combustible gases such as NH_3_ [27]. The intensity of several wood-derived pyrolysis bands was weaker in the plywood than in the untreated veneer, suggesting that the MF adhesive may partially dilute combustible volatiles and delay the thermal decomposition of the plywood structure.

The incorporation of EG further altered the pyrolysis gas profile. In the 200EG group, the intensities of C–H, C–O, and C–O–C absorption bands were generally reduced after EG addition, indicating that EG expansion hindered thermal degradation and volatile release. As shown in Figure 4C,D, at 10–20 phr, the CO_2_ and CO-related signals at 400–700 °C were lower than those of the control plywood. This suggests that moderate EG loading promoted the formation of an expanded protective char layer with relatively continuous apparent coverage, which reduced heat transfer and suppressed the release of pyrolysis gases from the underlying wood. However, when the EG content increased to 30–40 phr, the CO_2_ and CO-related absorption intensities increased at 400–700 °C, as shown in Figure 4E,F. This result indicates that excessive EG loading may weaken the gas-suppression effect under pyrolysis conditions. Several factors may explain this phenomenon. First, high EG contents can lead to excessive expansion and the formation of a visually loose or discontinuous char surface. Such discontinuous regions may provide additional pathways for the escape of pyrolysis gases. Second, a high filler loading can reduce the integrity of the adhesive layer and plywood structure, promoting crack formation during heating. Third, the decomposition of EG intercalating agents may generate additional gaseous products, including CO_2_, H_2_O, SO_2_, and sulfate-related species, thereby contributing to stronger FTIR signals at elevated temperatures [25,28,29].

The 450EG group exhibited a similar trend. As shown in Figure 4H,I, 450EG at 10–20 phr reduced the intensity of wood-derived pyrolysis gas bands, indicating that the higher expansion ratio could provide an effective thermal barrier at moderate loading levels. However, at 40 phr, the CO_2_ and CO-related signals increased markedly at 400–700 °C, as shown in Figure 4K. This finding suggests that high-expansion-ratio EG may become less effective when used at excessive contents because intense expansion can disrupt plywood structure and generate a visually less continuous char surface, facilitating volatile release. The FTIR results therefore reveal a complex relationship between EG content, expansion ratio, char-layer structure, and pyrolysis gas evolution. Moderate EG loading can suppress thermal decomposition by forming a protective barrier, whereas excessive EG loading or overly high expansion may produce discontinuous char regions, promote structural cracking, and enhance gas release during high-temperature decomposition. These findings are consistent with the cone calorimeter and char morphology results, which showed that EG improved heat-release performance but required optimization to maintain apparent char-layer continuity and plywood mechanical performance.

## 4. Conclusions

This study demonstrated that incorporating expanded graphite (EG) into melamine–formaldehyde (MF) resin is a feasible adhesive-based strategy for improving the fire-retardant performance of Japanese cedar plywood. EG expansion ratio and content strongly influenced the balance between physicomechanical properties and fire-retardant efficiency. EG incorporation had limited effects on plywood density and moisture content, and all panels satisfied the basic requirements for ordinary plywood. However, increasing EG content generally reduced bonding shear strength and parallel-direction flexural properties, particularly in the 450EG series. Under the present processing conditions, this reduction was mainly associated with the larger particle size and stronger expansion behavior of 450EG, which may have promoted poorer dispersion, void formation, and discontinuities within the wood–adhesive interphase. Cone calorimeter analysis showed that EG effectively suppressed heat release by forming an expanded carbonaceous char layer that acted as a thermal and mass-transfer barrier. Although none of the plywood formulations fully met the CNS Grade 3 fire-retardant requirement because their THR values remained above the specified limit, EG addition substantially reduced HRR and THR compared with the control plywood. It should also be noted that the THR values reported in this study represent the cumulative heat release within the 300 s test duration specified by the cone calorimeter protocol and do not represent the total heat release after complete combustion or flame extinction. Therefore, future studies should include extended cone calorimeter measurements and time-to-extinction analysis to provide a more comprehensive assessment of residual fire hazards. Among the tested formulations, 200EG_30_ provided the most favorable overall performance, combining reduced heat release, visually improved char coverage, and acceptable mechanical properties. High-temperature furnace–FTIR analysis further indicated that moderate EG loading suppressed the release of wood-derived volatile products, whereas excessive EG content or high-expansion-ratio EG increased CO_2_ and CO-related signals at 400–700 °C. Overall, these findings suggest that, under the present plywood manufacturing conditions, 30 phr 200EG is a promising baseline formulation for the modification and performance optimization of halogen-free fire-retardant wood-based composite panels.

## Figures and Tables

**Table 1:** Effects of EG expansion ratio and content on the density, moisture content, bonding shear strength, MOE, and MOR of Japanese cedar plywood.

Plywood	Density (kg/m^3^)	Moisture Content (%)	Bonding Shear Strength (kPa)		MOR (MPa)		MOE (GPa)	
			Mean	Minimum	*∥*	⊥	*∥*	⊥
200EG_0_	534 ± 24 ^a^	8.5 ± 0.3 ^c^	799 ± 83 ^a^	673	63 ± 6 ^a^	12 ± 2 ^a^	7.4 ± 0.5 ^a^	0.7 ± 0.1 ^a^
200EG_10_	478 ± 19 ^c^	8.9 ± 0.5 ^bc^	573 ± 92 ^b^	434	48 ± 6 ^b^	9 ± 1 ^b^	6.2 ± 0.5 ^b^	0.7 ± 0.1 ^a^
200EG_20_	493 ± 20 ^bc^	9.9 ± 0.6 ^a^	604 ± 75 ^b^	486	51 ± 8 ^b^	9 ± 1 ^b^	6.2 ± 0.7 ^b^	0.7 ± 0.0 ^a^
200EG_30_	525 ± 27 ^ab^	9.4 ± 0.3 ^ab^	508 ± 43 ^b^	446	47 ± 7 ^b^	10 ± 1 ^b^	5.7 ± 1.1 ^b^	0.7 ± 0.1 ^a^
200EG_40_	531 ± 39 ^a^	8.5 ± 0.6 ^c^	538 ± 42 ^b^	453	45 ± 10 ^b^	11 ± 3 ^ab^	7.0 ± 0.7 ^ab^	0.8 ± 0.2 ^a^
450EG_0_	543 ± 28 ^B^	10.5 ± 0.8 ^A^	799 ± 83 ^A^	673	56 ± 12 ^A^	12 ± 2 ^B^	7.4 ± 0.9 ^A^	0.8 ± 0.1 ^B^
450EG_10_	608 ± 18 ^A^	10.4 ± 0.7 ^A^	797 ± 36 ^A^	751	41 ± 12 ^A^	12 ± 1 ^B^	5.3 ± 1.5 ^B^	0.7 ± 0.1 ^B^
450EG_20_	591 ± 38 ^A^	9.0 ± 0.1 ^C^	522 ± 90 ^B^	439	38 ± 20 ^AB^	14 ± 4 ^B^	5.1 ± 2.1 ^B^	1.0 ± 0.3 ^AB^
450EG_30_	533 ± 17 ^B^	10.0 ± 0.6 ^AB^	518 ± 76 ^B^	383	40 ± 20 ^AB^	15 ± 6 ^A^	4.7 ± 3.4 ^B^	1.0 ± 0.5 ^AB^
450EG_40_	576 ± 51 ^AB^	9.5 ± 0.4 ^BC^	470 ± 121 ^B^	253	26 ± 8 ^B^	14 ± 2 ^B^	3.8 ± 1.5 ^B^	1.0 ± 0.2 ^A^

Values for density, moisture content, and bonding shear strength are expressed as the mean ± SD (*n* = 11). Values for MOE and MOR are expressed as the mean ± SD (*n* = 13). The symbols ***∥*** and **⊥** indicate that the face-grain direction was parallel and perpendicular to the span direction, respectively. Lowercase and uppercase letters indicate statistical comparisons within the 200EG and 450EG series, respectively. Different letters within the same column indicate significant differences among groups at *p* < 0.05.

**Table 2:** Effects of EG expansion ratio and content on HRR_peak_ and THR_300s_ of Japanese cedar plywood.

Plywood	HRR_peak_ (kW/m^2^)	THR_300s_ (MJ/m^2^)
200EG_0_	189 ± 3 ^a^	32.7 ± 1.4 ^a^
200EG_10_	197 ± 23 ^a^	23.2 ± 7.0 ^ab^
200EG_20_	180 ± 7 ^a^	16.2 ± 2.5 ^b^
200EG_30_	157 ± 23 ^a^	12.9 ± 0.8 ^b^
200EG_40_	196 ± 2 ^a^	13.0 ± 0.9 ^b^
450EG_0_	318 ± 40 ^A^	46.2 ± 2.6 ^A^
450EG_10_	179 ± 13 ^B^	32.3 ± 5.7 ^B^
450EG_20_	190 ± 3 ^B^	25.6 ± 2.7 ^BC^
450EG_30_	187 ± 22 ^B^	15.1 ± 3.8 ^C^
450EG_40_	171 ± 6 ^B^	16.5 ± 2.4 ^C^

Values are expressed as the mean ± SD (*n* = 3). Lowercase and uppercase letters indicate statistical comparisons within the 200EG and 450EG series, respectively. Different letters within the same column indicate significant differences among groups at *p* < 0.05.

## Data Availability

The data presented in this study are available on request from the corresponding author.

## Supplementary Materials

The following supporting information can be downloaded at: https://www.mdpi.com/article/10.3390/polym18141710/s1, Table S1: Effect of resin spread rate on adhesive layer coverage at an EG content of 40 phr; Table S2: Effect of resin spread rate on the bonding shear test of plywood (with 40 phr EG); Table S3: Effect of EG content and expansion ratios on adhesive viscosity; Figure S1: Surface images of veneers prepared with the addition of 40 phr of EG (expansion ratios of 200 and 450) under resin spread rates of 150 (A), 200 (B), 250 (C), 300 (D), and 350 g/m^2^ (E).
