# Supplementary material for: Expanded Graphite-Modified Melamine–Formaldehyde Adhesive for Fire-Retardant Japanese Cedar Plywood: Physicomechanical and Combustion Performance
Source: Polymers (Basel). 2026 Jul 12;18(14):1710. doi: 10.3390/polym18141710 (PMC13416723; doi:10.3390/polym18141710)
Supplement: Supplementary file 1 [file polymers-18-01710-s001.zip › polymers-4424831-supplementary.pdf]

Supplementary data

Table S1. Effect of resin spread rate on adhesive layer coverage at an EG content of 40 phr.

Sample code	Resin spread rate (g/m^2)	EG coverage ratio (%)	Coefficient of variation
200EG	150	92.2 ± 1.2^{b}	0.01
	200	96.4 ± 1.8^{a}	0.02
	250	98.8 ± 1.1^{a}	0.01
	300	99.8 ± 0.2^{a}	0.00
	350	99.9 ± 0.1^{a}	0.00
450EG	150	83.3 ± 2.0^{C}	0.02
	200	89.3 ± 1.7^{B}	0.02
	250	99.4 ± 0.5^{A}	0.01
	300	99.9 ± 0.0^{A}	0.00
	350	99.9 ± 0.1^{A}	0.00

The EG coverage ratio in the adhesive layer was quantified from digital images using ImageJ software (National Institutes of Health, Bethesda, MD, USA) with a fixed-threshold binarization method. Values are presented as mean ± SD (n = 3). Lowercase and uppercase letters indicate statistical comparisons within the 200EG and 450EG series, respectively. Different letters within the same column indicate significant differences among groups at $p < 0.05$.

Table S2. Effect of resin spread rate on the bonding shear test of plywood (with 40 phr EG).

Sample code	Resin spread rate (g/m^2)	Bonding shear strength	
		Mean (kPa)	Minimum (kPa)
200EG	150	511 ± 36[a]	465
	200	497 ± 62[a]	379
	250	468 ± 52[a]	404
	300	538 ± 42[a]	453
	350	468 ± 70[a]	331
450EG	150	572 ± 59[A]	483
	200	435 ± 159[A]	74
	250	204 ± 125[B]	0
	300	162 ± 156[B]	0
	350	411 ± 169[A]	0

Values are presented as mean ± SD (n = 11). Lowercase and uppercase letters indicate statistical comparisons within the 200EG and 450EG series, respectively. Different letters within the same column indicate significant differences among groups at $p < 0.05$.

Table S3. Effect of EG content and expansion ratios on adhesive viscosity.

Sample code	Viscosity (cps)
MF resin	$527 \pm 21^{d, D}$
$200EG_{10}$	668 ± 53^{d}
$200EG_{20}$	1544 ± 191^{c}
$200EG_{30}$	7420 ± 24^{b}
$200EG_{40}$	11059 ± 15^{a}
$450EG_{10}$	870 ± 118^{D}
$450EG_{20}$	1369 ± 188^{C}
$450EG_{30}$	2954 ± 68^{B}
$450EG_{40}$	4380 ± 34^{A}

Values are presented as mean ± SD ($n = 3$). Lowercase and uppercase letters indicate statistical comparisons within the 200EG and 450EG series, respectively. Different letters within the same column indicate significant differences among groups at $p < 0.05$.

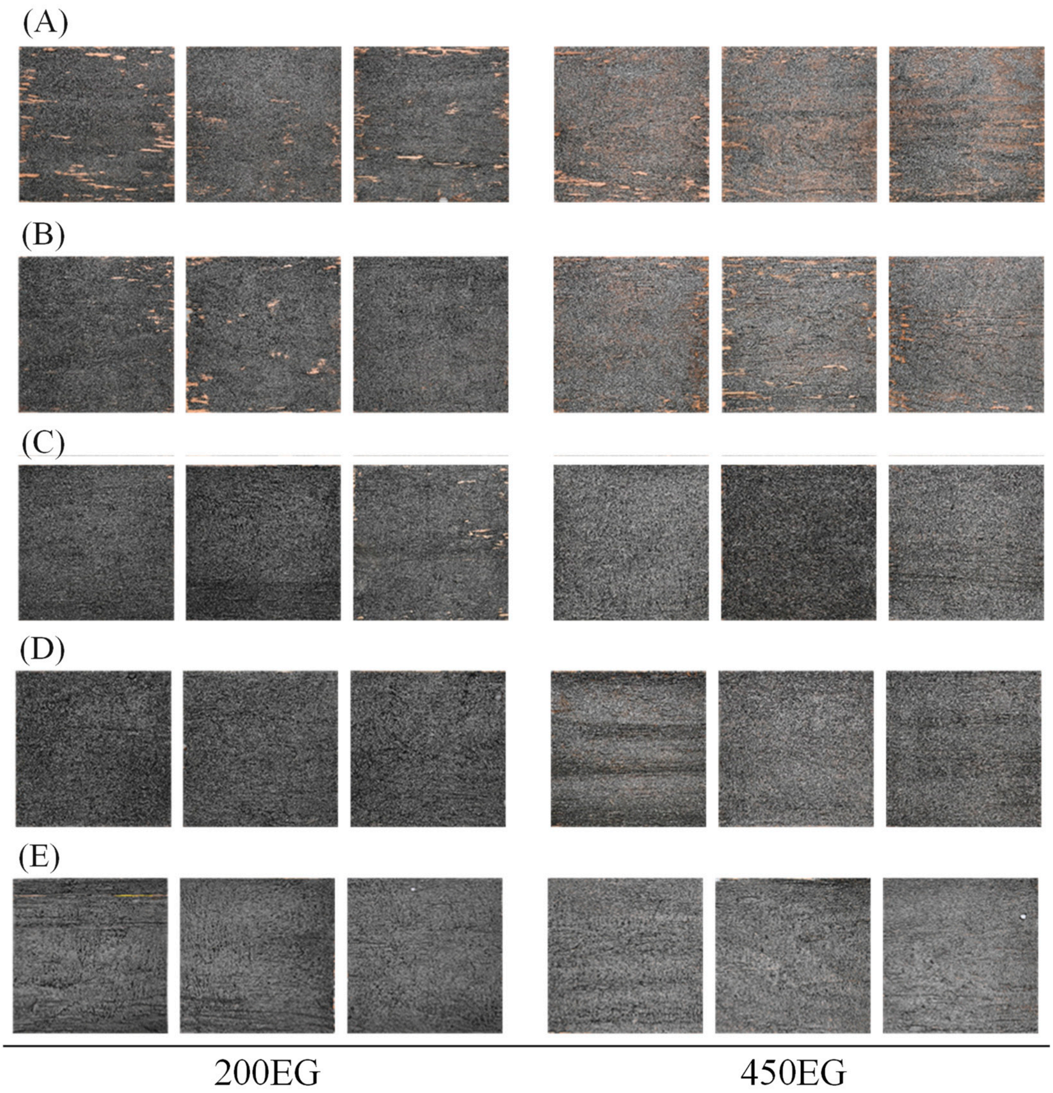

Figure S1. Surface images of veneers prepared with the addition of 40 phr of EG (expansion ratios of 200 and 450) under resin spread rates of 150 (A), 200 (B), 250 (C), 300 (D), and 350 g/m² (E).
